# Dual-Function Analysis of Astaxanthin on Golden Pompano (*Trachinotus ovatus*) and Its Role in the Regulation of Gastrointestinal Immunity and Retinal Mitochondrial Dysfunction Under Hypoxia Conditions

**DOI:** 10.3389/fphys.2020.568462

**Published:** 2020-12-01

**Authors:** Jin Niu, Wei Zhao, Dan-Qi Lu, Jia-Jun Xie, Xuan-Shu He, Hao-Hang Fang, Shi-Yu Liao

**Affiliations:** State Key Laboratory of Biocontrol, Institute of Aquatic Economic Animal and Guangdong Province Key Laboratory for Aquatic Economic Animals, School of Life Sciences, Sun Yat-sen University, Guangzhou, China

**Keywords:** astaxanthin, *Trachinotus ovatus*, gut morphology and microbiota, eyeball morphology, retina, mitochondrial

## Abstract

The present study investigated the potential mechanisms of astaxanthin in the regulation of gastrointestinal immunity and retinal mitochondrial function of golden pompano (*Trachinotus ovatus*). Triplicate groups of juvenile *T. ovatus* (mean initial weight: 6.03 ± 0.01 g) were fed one of six diets (D1, D2, D3, D4, D5, and D6) for 8 weeks, with each diet containing various concentrations of astaxanthin (0, 0.0005, 0.001, 0.005, 0.01, or 0.1%, respectively). Growth performance of fish fed the D2–D5 diets was higher than that of fish fed the D1 diet; however, growth performance and survival of fish deteriorated sharply in fish fed the D6 diet. Gut villus in fish fed the D2–D5 diets were significantly longer and wider than that of fish fed the D6 diet. Feeding with D2–D5 diets led to increased abundance of *Bacillus*, *Pseudomonas*, *Oceanobacillus*, *Lactococcus*, *Halomonas*, *Lactobacillus*, and *Psychrobacter* while abundance of *Vibrio* and *Bacterium* decreased. Additionally, feeding with the D6 diet resulted in a sharp decline in *Pseudomonas* and *Lactobacillus* abundance and a sharp increase in *Vibrio* abundance. A low dissolved oxygen environment (DO, 1.08 mg/L) was conducted for 10 h after the rearing trial. No fish mortality was observed for any of the diet treatments. Lysozyme (LZY) activity in fish fed the D6 diet decreased sharply and was significantly lower than that in other groups. ROS production also decreased sharply in fish fed the D6 diet. Moreover, the conjunctiva and sclera in the fish fed the D6 diet were indistinguishable. Suitable dietary astaxanthin supplementation levels (0.005–0.1%) exerting a neuroprotective effect from low dissolved oxygen environments is due to up-regulated expression of anti-apoptotic factors, such as phosphorylated Bcl-2-associated death promoter (pBAD), phosphorylated glycogen synthase kinase-3β (pGSK-3β), Bcl-2 extra large (Bcl-xL), and down-regulated expression of Bcl-2-associated X protein (Bax) pro-apoptotic factor in retinas. Furthermore, suitable dietary astaxanthin levels (0.0005–0.01%) suppressed up-regulation of critical mitochondrial components, such as peroxisome proliferator-activated receptor gamma coactivator-1α (PGC-1α), mitochondrial transcription factor A (TFAM), and mitochondrial DNA (mtDNA), while excessive astaxanthin supplementation produces the opposite effect. In brief, high-dose astaxanthin arouses and aggravates low dissolved oxygen-induced inflammation, oxidative stress, intestinal disorder, retinal apoptosis, and retinal mitochondrial dysfunction in *T. ovatus*. Second-degree polynomial regression of WG indicated that the optimum dietary astaxanthin for juvenile *T. ovatus* is 0.049%.

## Introduction

Aquaculture is the fastest-growing source of food production worldwide due to increased demand and decline of wild capture fisheries. However, many species of marine fish lose their natural skin coloration under captive conditions compared to the wild conditions. Reduced skin pigmentation in intensively cultured fish is associated with significant reductions in perceived quality of the fish and therefore yields lower market prices (Song et al., 2017). Carotenoids can largely enhance fish skin and muscle pigmentation but are not able to be synthesized by the body; therefore, carotenoids must be obtained through diet (Storebakken and No, 1992).

Carotenoids have been shown to promote growth performance and reproduction, improve antioxidant activity and immunity, and positively benefit intermediary metabolism and coloring of aquatic animals (Chuchird et al., 2015; Rahman et al., 2016; Song et al., 2017). Astaxanthin (3,3′-dihydroxy-β, β-carotene-4,4′dione) has stronger antioxidant activity than canthaxanthin and β-carotene, which are found in many microorganisms, marine animals (such as shrimp), microalgae, and yeast (Chatzifotis et al., 2005; Niu et al., 2012, 2014). Over the past decade, astaxanthin has also attracted great interest as novel replacement for antibiotics when used as an animal feed additive. Astaxanthin has few concerning adverse effects compared to antibiotic use and is generally considered safe in the aquafeed industry (Shimidzu et al., 1996).

Oxidative stress is caused by excessive production of reactive oxygen species (ROS), which can damage the function of cells and tissues (Dore, 2005). Astaxanthin is known for its ability to inhibit single ROS and to prevent oxidative stress, disease, and age-related degradation (Yang et al., 2013). Astaxanthin has reportedly prevented oxidative stress in proximal tubular epithelial cells by scavenging ROS from the mitochondria of mesangial cells, thus preventing diabetic nephropathy (Kim et al., 2009). Moreover, it is considered one of the most effective carotenoids for preventing various types of eye damage (Piermarocchi et al., 2012).

Astaxanthin has also been reported to exert beneficial effects on the gastrointestinal tract. *Haematococcus pluvialis*-derived astaxanthin was found to protect against gastric mucosal damage by activating antioxidative mechanisms *in vitro* (Kamath et al., 2008). Additionally, astaxanthin ester pretreatment has potentially protective effects against gastric mucosal injury and gastric acid secretion in rats (Kim et al., 2005a, b). The gastrointestinal tract of fish is considered an ecological niche and selects for beneficial bacteria from water sediment and feed (Burr et al., 2005). Moreover, microbial community diversity is related to gastrointestinal system function (Nayak, 2010). In fact, dietary immunostimulants have been shown to benefit host growth by improving intestinal microbial balance via structural and functional modifications of the fish gastrointestinal tract (Akter et al., 2016).

Golden pompano (*Trachinotus ovatus* Linnaeus 1758), an omnivorous–carnivorous marine fish, is widely cultured in China and other Asian countries due to its delicious meat and high nutritional value (Niu et al., 2013). However, when golden pompano are cultured under intensive farming conditions with seawater temperatures above 28°C, the fish often experience loss of their naturally occurring luminous yellow skin coloration, mild suppurative fluid accumulation in the intestines, reduced growth performance, decrease of immune ability, and eventually death, bringing enormous economic losses. These symptoms are collectively termed “summer gut syndrome” and can be immediately alleviated with antibiotics treatment; however, antibiotics can no longer be used due to international food security regulations issued by the Food and Agriculture Organization. Finding an alternative treatment that alleviates or cures summer gut syndrome in compliance with legal regulations is an urgent issue. One hypothesized cause of the gut syndrome is intolerance to intestinal microbiota composition, which manifests as reduced immunity. As the most powerful antioxidant in nature, astaxanthin has been proven to protect against potential oxidative stress by quenching or scavenging ROS (Kamath et al., 2008). No adverse effects have been found when astaxanthin has been used as a dietary supplement (Marazzi et al., 2011). The addition of astaxanthin can improve body color and immunity of aquatic animals. Although studies on the beneficial effects of astaxanthin in aquatic animals have also been published (Niu et al., 2012, 2014; Xie et al., 2017, 2020), additional well-designed gradient trials that include excessive astaxanthin levels are required to thoroughly understand potential mechanisms of astaxanthin. The results of the present experiment will offer new insight into nutraceutical supplements for people. Therefore, the present study was conducted to determine the effects and mechanism of dietary astaxanthin levels as it relates to golden pompano growth performance and antioxidation.

## Materials and Methods

### Ethics Statement and Study Design

All experimental and animal care procedures including the low dissolved oxygen stress tolerance test were approved by the Sun Yat-sen University, Guangzhou, China, and conformed to the National Institutes of Health Guide for Care and Use of Laboratory Animals (publication no. 85-23, revised 1985).

Six isonitrogenous and isoenergetic diets (D1, D2, D3, D4, D5, and D6) were formulated with six graded levels of astaxanthin. The basal diet (D1) formulation and proximate composition analysis are shown in Supplementary Table S1 and Table 1. The astaxanthin source Carophyll Pink (10% astaxanthin and 10% β-carotene, DSM Nutritional Products France SAS) was added to the basal diet of groups D2–D6 at concentrations of 0.005, 0.01, 0.05, 0.1, and 1%, respectively. Correspondingly, the supplementation of dietary astaxanthin in D1–D6 was 0%, 0.0005, 0.001, 0.005, 0.01, and 0.1%, respectively.

**TABLE 1:** Formulation and proximate composition of each diet (% dry matter)^1^.

	D1	D2	D3	D4	D5	D6
Basal diet	100	99.995	99.99	99.95	99.9	99
Carophyll Pink (10% astaxanthin)	0	0.005	0.01	0.05	0.1	1
Proximate composition						
Moisture	8.28	8.12	8.18	8.20	8.18	8.20
Crude protein	40.63	40.45	40.65	40.39	40.54	40.62
Crude lipid	13.10	13.06	13.29	13.19	13.16	13.16
Ash	9.52	9.47	9.57	9.48	9.52	9.55
Carotenoids (mg/g)	0.0033	0.0083	0.0135	0.0534	0.1034	1.0025

*^1^Values are means ± SEMs. *n* = 3 (one-factor ANOVA, Duncan’s multiple range test).*

### Experimental Procedures

The feeding trial was conducted out at an experimental station of South China Sea Fisheries Research Institute of CAFS (Sanya, Hainan). The fish were obtained from a commercial farm near Hongsha Bay, Sanya city, Hainan province, China. Prior to the feeding trial, the fish were reared in floating sea cages (3.0 m × 3.0 m × 3.0 m), and fed the control diet (D1) for 2 weeks to acclimate to the experimental diet and conditions. At the start of the experiment, the fish were fasted for 24 h and weighed after being anesthetized with tricaine methanesulfonate (1:30,000; Sigma-Aldrich Co., United States). The total number of 540 juvenile pompano (*T. ovatus*) with uniform size (initial body weight 6.03 ± 0.01) were randomly allotted into 18 sea cages (1.0 m × 1.0 m × 1.5 m; 3 cages per treatment), and each cage was stocked with 30 fish. Fish were fed by hand twice daily at 08:00–8:30 am and 16:00–16:30 pm, respectively. Fish were hand-fed slowly little by little to prevent waste of dietary pellets. When the experimental diet was supplied, the fish would swim to the water surface to ingest the diet. As long as fish were fed to satiation, they would never come up the water surface again. Hence, their apparent satiation could be judged by feeding behavior observation. The feeding trial lasted for 56 days. During the experimental period, feed consumption was recorded. Water temperature was maintained between 28 and 31°C. Water quality was maintained as follows: dissolved oxygen, >8.33 mg/L; salinity, 28–31 g/L. At the end of the experiment, the fish were fasted for 24 h before being anesthetized with tricaine methanesulfonate (1:30,000; Sigma-Aldrich Co., United States) and then fish in each cage were weighed and sampled. No welfare-related interventions were carried out prior to and during the experiment.

### Sample Collection and Chemical Analysis After the Rearing Trial

After 8 weeks of feeding, color chromatism of the gill cover, dorsal skin, ventral skin, dorsal fin, anal fin, and tail fin of three fish from each cage were measured using a WSC-S colorimeter (Shanghai Precision Scientific Instrument Co., Ltd., Shanghai, China). The lightness (L^∗^), redness (a^∗^), and yellowness (b^∗^) values were used to determine the degree of color change in each sample.

As for the intestinal histological examination, the fish were fasted for 24 h before being anesthetized with tricaine methanesulfonate (1:30,000; Sigma-Aldrich Co. United States) and then weighed at 8:30 am. The distal intestinal segments of two randomly selected fish from each cage were aseptically stripped and fixed in 4% paraformaldehyde for intestinal morphology analysis. The fixed distal intestinal segments were dehydrated in alcohol (30–100%) and embedded in paraffin. Tissue sections (1 μm thick) were stained with hematoxylin–eosin and intestinal morphology was observed with a light microscope (Leica DMLB, Germany). Villus length and width were measured according to the published method of Davenport (Davenport, 1969).

As for the DNA extraction from gut samples, the whole intestine was aseptically stripped from two randomly selected fish from each cage. Total bacterial DNA from the gut was extracted using the E.Z.N.A.^®^ Stool DNA Kit (Omega Biotek, Norcross, GA, United States), according to the manufacturer’s instructions.

### HPLC Conditions for Astaxanthin Analysis

Astaxanthin was analyzed by high-performance liquid chromatography (HPLC), using a Hitachi L-6200 pump, a silica column (Lichrosorb Si-60 5 micro 250 × 4.6 mm column I.D., E. Merck), a Hitachi L-4250 UV–VIS detector at 470 nm, and a Hitachi D-2000 Chromato-Integrator. The operational conditions were as follows: mobile phase, 14% acetone in n-hexane; solvent flow rate, 1.5 ml/min; injection volume, 100 μl; and pump program, the sequence was 0–20 min Mixture A and 20.5–40 min Mixture B; Mixture A was acetone/*n*-hexane, 14/86, and Mixture B was *n*-heptane 100%. This system was controlled by a chromatographic data system (Scientific Information Services), which also integrated the areas under the peaks. The astaxanthin standard was chromatographically pure astaxanthin and purchased from Sigma. Sample concentrations were integrated against external standards of known concentrations measured using a V530 UV/Vis spectrophotometer [Jasco (UK) Ltd., Great Dunmow, Essex, United Kingdom], with an extinction coefficient of E1%, 1 cm = 2100 at an absorbance maximum (λmax = 470 nm) for astaxanthin (Shiau et al., 1991).

Tissue carotenoids and astaxanthin were expressed on a dry weight basis to eliminate potential error resulting from variations in fish moisture content associated with different stages of development.

### Microbial Community Analysis

The V4 region of bacterial 16S rDNA was amplified using primer pair 515F (GTGCCAGCMGCCGCGGTAA) and 806R (GGACTACHVGGGTWTCTAAT). The reverse primer contained a 6-bp error-correcting barcode unique to each sample. DNA was amplified using the method described by Qin et al. (2010). Sequencing was performed using the Illumina MiSeq platform by Novogene (Beijing, China).

FLASH was used to merge the pairs of readings from the original DNA fragments (Magoè and Salzberg, 2011). QLIME software package^1^ and UPARSE pipeline^2^ were used to analyze the readings and pick operational taxonomic units (OTUs). The high-quality sequences were assigned to OTUs at 97% sequence similarity and then classified taxonomically using the Ribosomal Database Project (RDP) classifier (Wang et al., 2007). The raw readings were deposited into the NCBI Sequence Read Archive (SRA) database (Accession Number: SRP212981).

Alpha diversity indices, including abundance-based coverage estimators (ACE), Simpson diversity index, Shannon diversity index, and Chao1 index (Chao1), were calculated by QIIME^3^ and used to assess microbiota diversity and uniformity.

### Low Dissolved Oxygen Stress Tolerance Test

*Trachinotus ovatus*, which naturally swims constantly in the water, is particularly very sensitive to lack of dissolved oxygen. We measured the effect of astaxanthin on the tolerance of *T. ovatus* to stress by reducing dissolved oxygen concentrations in fish enclosures. After the rearing trial, 15 fish per cage were randomly selected for a hypoxic stress test. The low dissolved oxygen (DO, 1.08 mg/L) condition was maintained by stopping aeration. Fish were stressed in this way during the daytime for 10 h (08.00–18.00) for 1 day. Survival in each chamber was recorded.

### Sample Collection and Chemical Analysis After the Low DO Stress Tolerance Test

As for the plasma lysozyme (LZY) analysis, six fish per diet treatment were selected randomly and anesthetized with tricaine methanesulfonate until death. Blood was drawn from the caudal vein into disposable syringes pre-rinsed with heparin sodium and then centrifuged for 15 min at 2000 *g* to collect the plasma. Plasma was stored at −80°C for biochemical analysis. Plasma LZY activity was measured by a turbidimetric assay according to the method described by Skouras et al. (2003).

As for the ROS analysis from the head kidney, the head kidney from each of the fish used in the plasma LZY analysis was placed in centrifuge tubes (50 ml) filled with 15 ml of wash medium (RPMI medium mixed with 10,000 IU sodium heparin; Sigma-Aldrich Co., United States). Generation of ROS by head kidney leukocytes (HKLs) was measured by a nitroblue tetrazolium salt (NBT) reduction assay as described by Skouras and Steinhagen (2003).

As for the relative mt DNA content measurement from the retinas, 12 fish per diet group were anesthetized with tricaine methanesulfonate and the eye cups were dissected out. Retinas were stripped together with the retinal pigment epithelial layer. The retinas were placed in liquid nitrogen (-196°C) and stored at -80°C until RNA isolation. The relative amount of mitochondrial DNA (mtDNA) was measured using long fragment PCR according to the method described by Wang et al. (2017). Total retinal DNA from six fish per group was isolated using the genomic DNA isolation kit (TaKaRa, Dalian, China). The forward primer, ATATTTTCACTGCTGAGTCCCGTGG, and the reverse primer, AATTTCGGTTGGGGTGACCTCGGAG, were used to amplify mitochondrial genomes.

### Quantitative Real-Time Polymerase Chain Reaction (qRT-PCR)

qRT-PCR analysis was done on the relative mRNA expressions of microphthalmia-associated transcription factor (MITFα) and tyrosinase (TYR) in the ventral skin, on the relative mRNA expressions of rhodopsin, phosphorylated glycogen synthase kinase-3β (pGSK-3β), phosphorylated Bcl-2-associated death promoter (pBAD), Bcl-2 extra large (Bcl-xL), Bcl-2-associated X protein (Bax), and peroxisome proliferator-activated receptor gamma coactivator-1α (PGC-1α), and on the retinal mitochondrial transcription factor A (TFAM).

Total skin RNA and retinal RNA were isolated from 12 fish per group using Trizol (Invitrogen) according to the manufacturer’s instruction. cDNA was synthesized according to the PrimeScript^TM^ RT reagent Kit with gDNA Eraser (TaKaRa, Dalian, China). The primer sequences used for qRT-PCR are shown in Supplementary Table S2.

qRT-PCR was performed according to the manufacturer’s recommendations using the Green Real-time PCR Master Mix (TaKaRa, Dalian, China). qRT-PCR consisted of denaturation at 95°C for 2 min, followed by 40 amplification cycles of 95°C for 15 s, annealing at 58°C for 15 s, and extension at 72°C for 30 s. The 2^–ΔΔ*CT*^ method was used to analyze mRNA expression levels (Zhao et al., 2017).

### Calculations and Statistical Analysis

Biological parameters used to evaluate the quality of diets were calculated by the following equations:

Weight gain (WG) (%) = 100 × (Wf - Wi)/Wi

Feed conversion ratio (FCR) = dry feed intake/(Wt - W0)

Survival (%) = 100 × Nt/N0

Wf and Wi were the mean final and initial fish body weights, respectively; Nt is the number of fish at the end of the feeding trial and N0 is the number of fish at the start of the feeding trial; Wt (g) is the total final body weight and W0 (g) is the total initial body weight.

The total carotenoid content per 100 g of tissue was calculated according to the following equation (McBeth, 1972):

mg carotenoids/100 g tissue = (OD × vol. × 103)/(E × TW)

where OD is the optical density at λmax (476 nm); vol. is the total volume of solution (ml); E is the absorption coefficient (the average coefficient of 2500 was usually used in the calculation); and TW is the weight of tissue (g).

Carotenoid retention efficiency in tissues (%) = 100 × (carotenoid content of final tissue - carotenoid content of initial tissue)/carotenoid intake.

Data are presented as means ± SEMs. All data were checked for normality and homogeneity of variance before analysis. Data on the direct effects of astaxanthin on golden pompano were subjected to one-factor ANOVA with Duncan’s multiple range test. All the data were analyzed using SPSS 19.0 software, and *P* < 0.05 was considered statistically significant. Quadratic regression analysis (Zeitoun et al., 1976) was used to determine the points in growth parameter, which represented the optimal dietary level of astaxanthin for *T. ovatus*.

### Statement of Adverse Events

No adverse events happened in each experimental group.

## Results

### Biological Performance

Fish biological performance is presented in Table 2. The growth performance (FBW and WG) of fish fed the D4 and D5 diets were significantly higher than that of fish fed the D1 and D6 diets (*P* < 0.05); no significant differences were found in growth performance among fish fed D2–D5 diets (*P* > 0.05). The feed conversion ratio (FCR) of fish fed the D4 diet was significantly lower than that of fish fed the D1, D2, and D6 diets (*P* < 0.05); there was no significant difference in FCR among fish fed D3–D5 diets (*P* > 0.05). Survival of fish fed the D6 diet was significantly lower than that of fish fed other diets (*P* < 0.05).

**TABLE 2:** Growth performance and survival of golden pompano fed diets with different levels of astaxanthin^1^.

Treatments	D1	D2	D3	D4	D5	D6
IBW, g/fish	6.01 ± 0.01	6.02 ± 0.01	6.04 ± 0.01	6.06 ± 0.02	6.03 ± 0.01	6.02 ± 0.02
FBW, g/fish	76.54 ± 0.26^a^	79.39±1.20^*ab*^	82.58±3.48^*ab*^	86.51 ± 1.33^b^	85.78 ± 1.61^b^	78.37 ± 3.16^a^
WG, ^2^ %	1174 ± 4.20^a^	1219±19.88^*ab*^	1267±55.33^*ab*^	1329 ± 23.15^b^	1323 ± 28.67^b^	1202 ± 52.31^a^
FCR ^3^	1.60 ± 0.31^b^*c*	1.50 ± 0.10^b^	1.39±0.06^*ab*^	1.21 ± 0.07^a^	1.33±0.08^*ab*^	1.76 ± 0.05^c^
Survival, ^4^ %	91.67 ± 6.01^b^	90.00 ± 5.00^b^	88.33 ± 3.33^b^	96.67 ± 3.33^b^	91.67 ± 4.41^b^	73.33 ± 1.67^a^

*^1^Values are means ± SEMs. *n* = 3 (IBW, FBW, WG, FCR, and Survival: replicates of 20 fish). Means in a row without a common superscript letter differ, *P* < 0.05 (one-factor ANOVA, Duncan’s multiple range test).FBW, final mean body weight; FCR, feed conversion rate; IBW, initial mean body weight; WG, weight gain.^2^WG = 100 × (FBW - IBW)/IBW.^3^FCR = dry food intake/wet weight gain.^4^Survival (%) = 100 × number of final fish/number of initial fish.*

**FIGURE 1:**
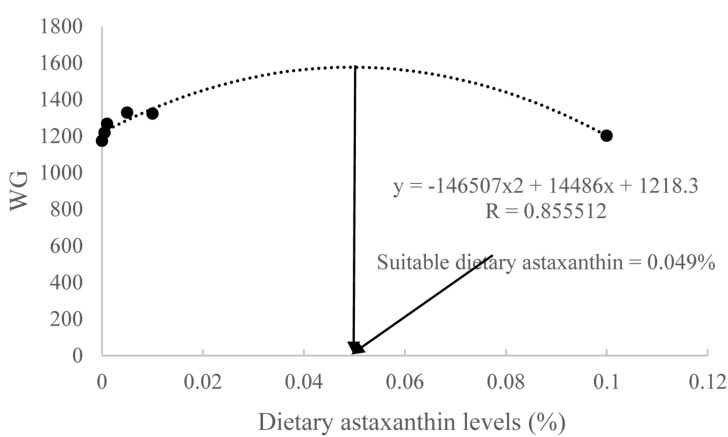
Second-degree polynomial relationship of weight gain (WG) to dietary astaxanthin levels.

When the quadratic regression analysis was used for estimating the optimum dietary astaxanthin for maximal growth of juvenile *T. ovatus*, the regression equations used was *y* = -146,507*x*^2^ + 14,486*x* + 1218.3 (Figure 1). The point of dietary astaxanthin at 0.049% was estimated to be the levels to provide maximal growth of juvenile *T. ovatus*.

### Color, Carotenoid Contents and Retention Efficiencies, and Expression Profile of Skin MITFα mRNA and TYR mRNA

**FIGURE 2:**
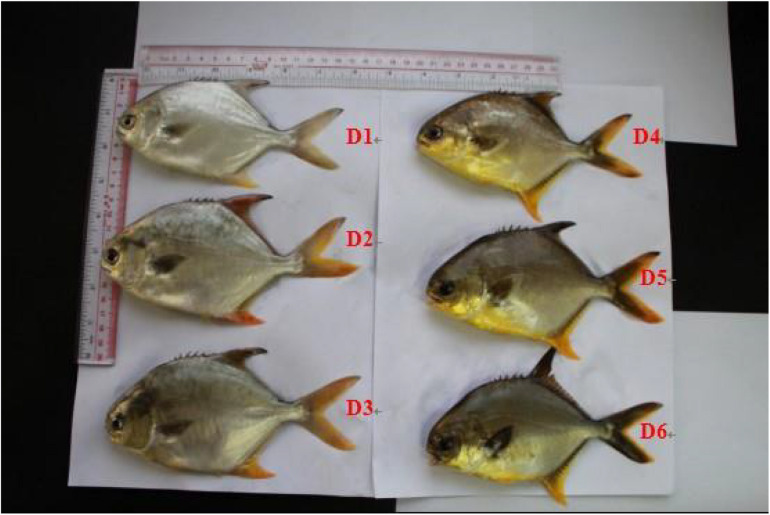
Color of live golden pompano fed the six experimental diets.

Differences in fish body coloration among diet treatments were first observed on week 2. Compared with the control group (D1), fish fed the astaxanthin-supplemented diets (D2–D6) exhibited more luminous yellow color (Figure 2). After the 8-week feeding trial, the luminous yellow coloring of fish from the D4–D6 diet groups was more apparent than that of fish from the D1–D3 diet groups.

Tissue color values are shown in Table 3. After the 8-week feeding trial, L^∗^ values of the ventral skin and fins in the D2–D6 groups were lower than those in the D1 group (*P* < 0.05). Dorsal fin a^∗^ value increased as concentration of dietary astaxanthin increased. Anal fin and tail fin a^∗^ values did not change significantly when dietary astaxanthin supplementation reached 0.005%. Dorsal skin, ventral skin, anal fin, and tail fin b^∗^ values plateaued when dietary astaxanthin supplementation reached 0.005%.

**TABLE 3:** Effects of different levels of astaxanthin on pigmentation of gill cover, skin, and fins in golden pompano^1^.

		D1	D2	D3	D4	D5	D6
L*	Gill cover	92.83 ± 2.33	91.46 ± 5.07	90.64 ± 4.68	92.26 ± 1.92	91.57 ± 2.21	92.21 ± 2.20
	Dorsal skin	68.36 ± 3.72	66.89 ± 2.88	66.12 ± 3.96	68.88 ± 4.68	67.71 ± 4.42	66.25 ± 4.54
	Ventral skin	95.72 ± 0.75^d^	95.39 ± 1.33^d^	94.85 ± 1.31^cd^	94.14 ± 1.61^bc^	93.51 ± 1.90^ab^	92.85 ± 1.74^a^
	Dorsal fin	50.15 ± 4.71^b^	46.29 ± 3.52^a^	45.04 ± 4.84^a^	43.35 ± 6.37^a^	44.88 ± 4.58^a^	46.11 ± 6.24^a^
	Anal fin	69.75 ± 2.14^b^	68.66 ± 4.13^ab^	67.77 ± 2.09^ab^	67.33 ± 2.72^a^	66.58 ± 2.84^a^	67.38 ± 3.16^a^
	Tail fin	66.92 ± 5.01^b^	64.67 ± 5.37^ab^	65.60 ± 5.90^ab^	65.41 ± 3.81^ab^	63.59 ± 2.22^ab^	62.64 ± 5.75^a^
a*	Gill cover	−1.72 ± 0.63	−1.31 ± 0.58	−1.78 ± 1.15	−1.93 ± 0.73	−1.74 ± 0.82	−1.55 ± 0.67
	Dorsal skin	−0.17 ± 1.22^a^	0.38 ± 0.70^ab^	0.31 ± 1.2^ab^	0.56 ± 1.13^ab^	0.47 ± 1.10^ab^	0.73 ± 0.78^b^
	Ventral skin	−2.31 ± 0.51^a^	−2.35 ± 0.67^a^	−1.94 ± 0.56^ab^	−1.19 ± 1.33^b^	−1.33 ± 1.15^b^	−1.45 ± 1.41^b^
	Dorsal fin	3.99 ± 0.59^a^	4.69 ± 0.43^b^	5.21 ± 1.01^b^	6.41 ± 1.09^cd^	6.88 ± 0.93^d^	6.01±*k*0.96^c^
	Anal fin	7.98 ± 1.51^a^	7.09 ± 1.61^a^	7.45 ± 1.15^a^	12.65 ± 2.11^b^	12.62 ± 2.25^b^	13.04 ± 1.95^b^
	Tail fin	5.06 ± 0.68^a^	5.71 ± 1.05^a^	5.87 ± 0.86^a^	9.45 ± 2.25^b^	9.11 ± 1.45^b^	8.63 ± 1.84^b^
b*	Gill cover	11.16 ± 4.05^a^	13.008 ± 4.54^a^	18.28 ± 3.37^b^	30.93 ± 7.29^c^	28.54 ± 6.79^cd^	25.29 ± 5.35^d^
	Dorsal skin	5.07 ± 2.09^a^	4.70 ± 1.85^a^	5.60 ± 1.51^a^	9.45 ± 2.38^b^	9.73 ± 3.05^b^	9.67 ± 1.98^b^
	Ventral skin	10.70 ± 5.60^a^	10.71 ± 8.19^a^	24.40 ± 10.30^b^	40.78 ± 14.65^c^	42.21 ± 13.43^c^	43.58 ± 11.72^c^
	Dorsal fin	13.07 ± 1.82^a^	15.19 ± 1.42^b^	17.61 ± 1.81^c^	19.36 ± 1.87^d^	24.44 ± 2.49^f^	20.98 ± 2.60^e^
	Anal fin	45.09 ± 4.35^a^	43.93 ± 6.31^a^	46.00 ± 5.66^a^	58.64 ± 9.16^b^	61.21 ± 6.80^b^	59.53 ± 5.75^b^
	Tail fin	30.47 ± 3.26^a^	29.55 ± 4.42^a^	31.57 ± 5.88^a^	42.40 ± 9.02^b^	45.26 ± 4.20^b^	43.64 ± 6.08^b^

*^1^Values are means ± SEMs. *n* = 3. Means in a row without a common superscript letter differ, *P* < 0.05 (one-factor ANOVA, Duncan’s multiple range test). L^∗^ value for lightness was ranging from 0 for black to 100 for white. a^∗^ value means red/green from 128 to -128. b^∗^ value indicates yellow/blue.*

Ventral muscle and ventral skin carotenoid and astaxanthin contents, and carotenoid retention efficiencies of fish are shown in Table 4. The ventral muscle and ventral skin carotenoid contents were increased with the increase dietary astaxanthin level until 0.005% and then reached a plateau, while the trend of astaxanthin content in ventral muscle was different from that of astaxanthin content in ventral skin. The ventral muscle astaxanthin content was increased with the increase of dietary astaxanthin level until 0.01% and then reached a plateau. The ventral skin astaxanthin content was increased with the increase of dietary astaxanthin level until 0.005% and then reached a plateau. Carotenoid retention efficiency decreased with increased dietary astaxanthin levels in the ventral muscle. Interestingly, a trend of carotenoid retention efficiency in the ventral skin first increasing and then decreasing was shown with the increase of dietary astaxanthin level. Therefore, carotenoid retention efficiency of the ventral skin was maximized when the fish were fed the D4 diet.

**TABLE 4:** Carotenoid content (mg/g), astaxanthin content (mg/g), and carotenoid retention efficiency (%) in ventral muscle and ventral skin of fish fed the experimental diets^1^.

Treatments	D1	D2	D3	D4	D5	D6
**Carotenoid contents**						
Ventral muscle (× 10^–4^, mg g^–1^)	31.61 ± 0.25^a^	34.08 ± 0.08^b^	44.06 ± 0.10^c^	69.81 ± 0.17^d^	69.57 ± 0.75^d^	70.39 ± 0.25^d^
Ventral skin (× 10^–4^, mg g^–1^)	91.00 ± 0.18^a^	101.3 ± 0.13^b^	118.3 ± 0.13^c^	294.3 ± 1.41^d^	293.8 ± 0.56^d^	294.2 ± 1.08^d^
**Astaxanthin contents**						
Ventral muscle (× 10^–4^, mg g^–1^)	15.07 ± 0.12^a^	18.41 ± 0.22^b^	23.32 ± 0.45^c^	33.20 ± 0.62^d^	34.77 ± 0.20^e^	35.71 ± 0.99^e^
Ventral skin (× 10^–4^, mg g^–1^)	31.00 ± 0.18^a^	41.34 ± 0.13^b^	58.32 ± 0.13^c^	84.28 ± 1.41^d^	83.77 ± 0.56^d^	84.23 ± 1.08^d^
**Carotenoid retention efficiency**						
Ventral muscle		32.60 ± 2.38^d^	30.63 ± 0.86^d^	12.04 ± 0.71^c^	6.30 ± 0.42^b^	0.67 ± 0.03^a^
Ventral skin		15.16 ± 1.16^b^	23.11 ± 0.61^c^	36.70 ± 2.35^d^	21.48 ± 0.89^c^	2.03 ± 0.07^a^

*^1^Values are means ± SEMs. *n* = 3. Means in a row without a common superscript letter differ, *P* < 0.05 (one-factor ANOVA, Duncan’s multiple range test).*

**FIGURE 3:**
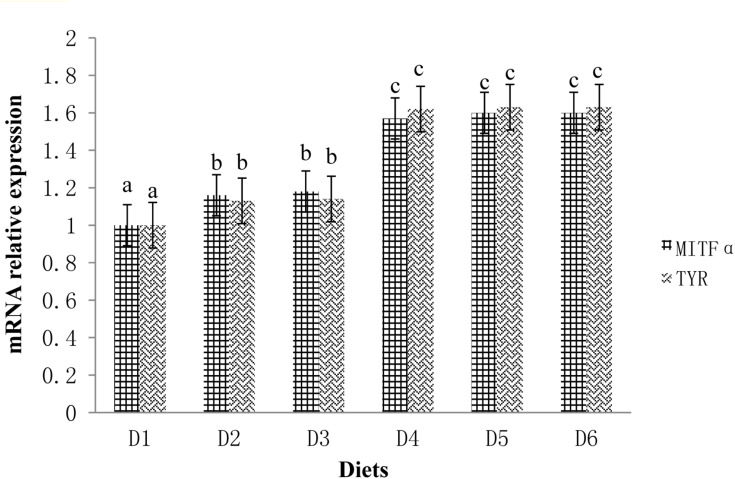
Relative mRNA expressions of MITFα and TYR in the ventral skin of golden pompano fed the six diets with different astaxanthin levels. Values are means ± SEMs of three replicates, with standard errors represented by vertical bars. ^*a, b, c*^ values in the vertical bar with different letters are significantly different (*P* < 0.05). The relative expression of MITFα mRNA is as follows: D1, 1.00 ± 0.01; D2, 1.16 ± 0.02; D3, 1.18 ± 0.02; D4, 1.57 ± 0.03; D5, 1.60 ± 0.01; D6, 1.60 ± 0.01. The relative expression of TYR mRNA is as follows: D1, 1.00 ± 0.01; D2, 1.13 ± 0.03; D3, 1.14 ± 0.01; D4, 1.62 ± 0.01; D5, 1.63 ± 0.01; D6, 1.63 ± 0.04.

The expression profiles of fish skin MITFα mRNA and TYR mRNA are shown in Figure 3. The expression levels of MITFα and TYR were significantly lower in fish fed the D1 diet compared to fish fed the astaxanthin-supplemented diets (D2–D6) (*P* < 0.05). Moreover, when astaxanthin supplementation concentration reached 0.005%, skin MITFα mRNA and TYR mRNA expression levels plateaued.

### Representative Intestinal Structure, Gut Morphology Parameters, and Gut Microbial Community

**FIGURE 4:**
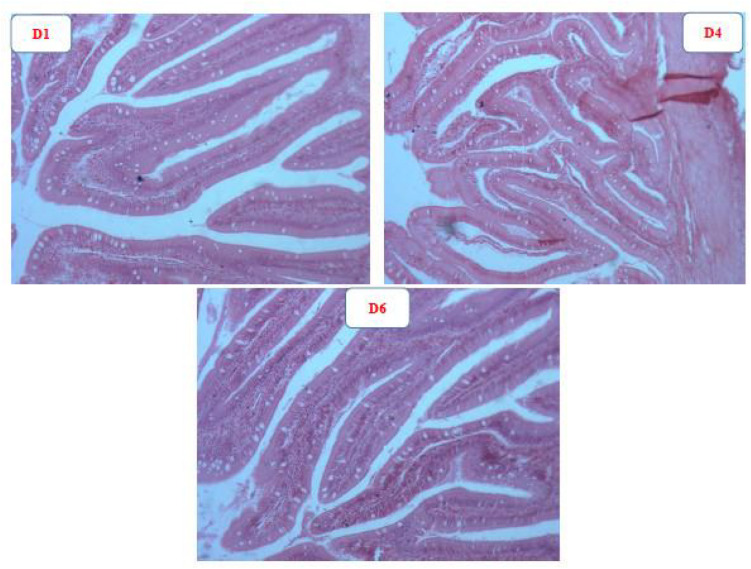
Representative intestinal structure of golden pompano fed D1, D4, and D6 diets.

The representative intestinal structure and gut morphology parameters are shown in Figure 4 and Table 5, respectively. The intestinal structure of fish fed D4 diet clearly exhibited more folds than that of fish fed the D1 and D6 diets. The gut villus length and villus width of fish from the astaxanthin-supplemented groups were significantly higher than those of fish from the control group (*P* < 0.05). There were no significant differences among D2–D5 diet treatments in fish gut villus length and villus width (*P* > 0.05); however, the gut villus length and villus width was significantly lower in fish fed D6 diet compared to those fed the D2–D5 diets (*P* < 0.05).

**TABLE 5:** Gut morphology changed for inclusion of dietary astaxanthin^1^.

Treatments	D1	D2	D3	D4	D5	D6
Villus length (μm)	330.5 ± 2.63^a^	524.9 ± 3.15^c^	531.5 ± 4.66^c^	535.4 ± 2.11^c^	531.9 ± 3.22^c^	433.8 ± 3.97^b^
Villus width (μm)	64.7 ± 2.02^a^	114.3 ± 2.26^c^	119.3 ± 0.56^c^	116.1 ± 2.21^c^	116.8 ± 0.32^c^	92.0 ± 1.65^b^
Microvillus length (μm)	0.92 ± 0.02^a^	1.51 ± 0.02^c^	1.50 ± 0.02^c^	1.51 ± 0.02^c^	1.49 ± 0.03^c^	1.27 ± 0.04^b^

*^1^Values are means ± SEMs. *n* = 3. Means in a row without a common superscript letter differ, *P* < 0.05 (one-factor ANOVA, Duncan’s multiple range test).*

The alpha diversity index of golden pompano gut microbiota fed the experimental diets is shown in Supplementary Table S3. A total of 2,076,436 high-quality sequence reads were obtained and clustered into OTUs of ≥97% identity. The number of OTUs detected in each sample ranged from 363 to 423, and Good’s coverage ranged from 88.78 to 96.44%. The principal coordinate analysis showed that the control group and the astaxanthin supplementation groups were clustered separately, suggesting that gut microbiota was significantly altered by dietary astaxanthin. Composition and relative abundance of bacterial communities in phylum, class, order, family, and genus level are shown in Supplementary Figure S1. At the phylum level, Firmicutes (52.83–69.16%) was the dominant phylum in all six diet treatments, followed by Proteobacteria (27.38–37.04%). At the class level, Bacilli (47.36–67.10%) was the dominant class in all six diet treatments, followed by Gammaproteobacteria (24.35–32.87%). At the order level, Bacillales (40.96–58.74%) was the dominant order in all six diet treatments, followed by Pseudomonadales (8.32–19.05%). At the family level, Bacillaceae (40.24–57.82%) was the dominant family in all six diet treatments, followed by Pseudomonadaceae (6.39–12.05%). At the genus level, *Bacillus* (34.54–50.15%) was the dominant genus in all six diet treatments, followed by *Pseudomonas* (6.31–13.05%). We also found that the abundances of *Pseudomonas* and *Lactobacillus* decreased sharply while *Vibrio* abundance increased sharply in fish fed the D6 diet.

### Changes to Microbial Community Composition

According to the rarefaction curve, rank abundance curve, Shannon curves, PCoA, and index of microbial community (Supplementary Figure S2 and Supplementary Table S3), abundance and diversity of intestinal microbiota in the control group (D1) were highest, while the astaxanthin supplementation groups (D2–D6) contained less diverse microbes. Moreover, clustering analysis of PCoA (Supplementary Figure S2A) showed that astaxanthin-supplemented groups (D2–D6) were clustered together and separated from the control group (D1). However, as concentration of dietary astaxanthin increased, the abundance and diversity of intestinal microbiota first increased before decreasing, with the D4 group being most similar to the control group (D1) in peak abundance and diversity of intestinal microbiota (Supplementary Figures S2B–D).

According to the composition and relative abundance of bacterial communities (Supplementary Figure S2), the present results show that Fusobacteria, Fusobacteriia, and Fusobacteriales were not found in the astaxanthin-supplemented groups at the phylum, class, and order levels, respectively. Moreover, at the order level, Vibrionales abundance decreased with suitable dietary astaxanthin supplementation (0.0005–0.01%) compared to the control group but was elevated with excess astaxanthin dietary supplementation (0.1%). At the family level, Vibrionaceae and Streptococcaceae abundance decreased with suitable dietary astaxanthin supplementation (0.0005–0.01%) compared to the control group, but were elevated with excess astaxanthin dietary supplementation (0.1%). At the genus level, *Vibrio* abundance decreased while *Pseudomonas* and *Lactobacillus* abundance increased with suitable dietary astaxanthin supplementation, compared to the control group (0.0005–0.01%). However, *Vibrio* abundance increased while *Pseudomonas* and *Lactobacillus* abundance decreased with excess astaxanthin supplementation (0.1%).

**FIGURE 5:**
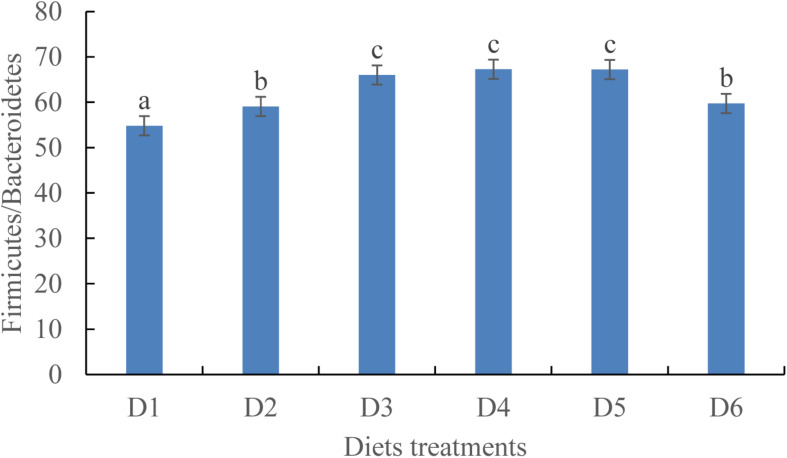
Ratio of *Firmicutes*/*Bacteroidetes* in phylum level among the six experimental diet treatments.

At the phylum level, the Firmicutes/Bacteroidetes ratio first increased before decreasing as dietary astaxanthin levels were incrementally increased. The Firmicutes/Bacteroidetes ratio of fish fed the diet highest in astaxanthin supplementation (D6) was significantly lower than that fed the control diet (Figure 5).

### Survival of Fish After the Low Dissolved Oxygen Tolerance Test

After 1 day of the low dissolved oxygen tolerance test, no mortality of fish was found among all the diet treatments.

### Plasma LZY Activity After the Low DO Stress Tolerance Test

**FIGURE 6:**
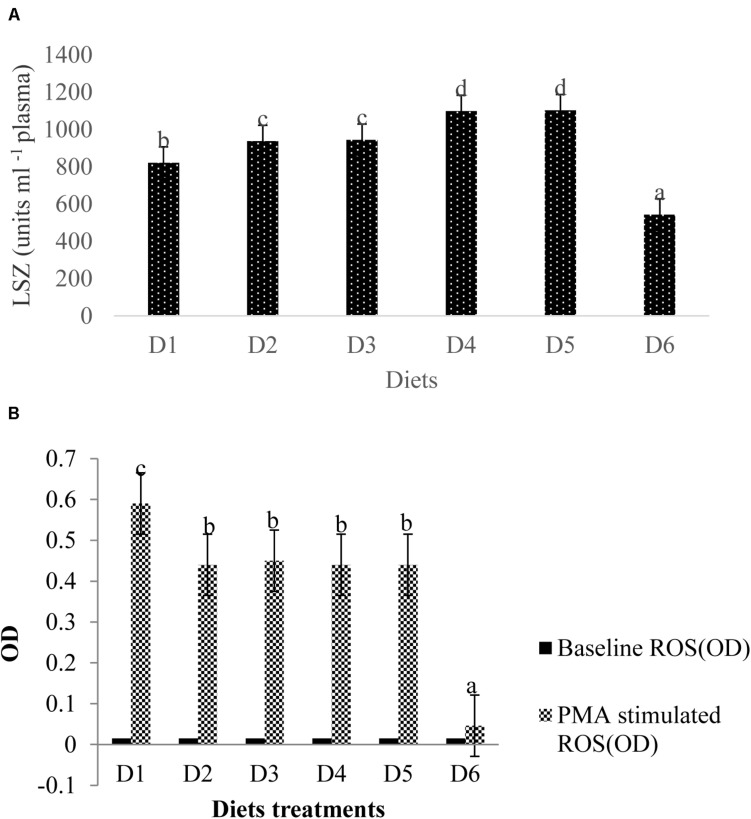
Analysis of immune-related index (**A:** LSZ activity in plasma; **B:** ROS level in head kidney) of fish. Different letters indicated significant differences between groups (*P* < 0.05). The LSZ activity is as follows: D1, 822 ± 13.58; D2, 937 ± 8.82; D3, 944 ± 6.08; D4, 1099 ± 6.12; D5, 1103 ± 9.13; D6, 543 ± 7.51. The values of the baseline ROS (OD) were the same of 0.015 ± 0.001 without significant differences (*P* > 0.05). The stimulated ROS values were measured between 0.046 ± 0.002 (D6) and 0.587 ± 0.012 (D1) with significant differences (*P* < 0.05).

Lysozyme activity measured in the blood plasma of golden pompano was altered by dietary astaxanthin levels (Figure 6A). LZY activity was significantly higher in fish fed D2–D5 diets compared to those fed the D1 diet (*P* < 0.05); however, LZY activity sharply declined by 543 ± 7.51 in fish fed the D6 diet compared to the fish fed a D1 diet, which had a LZY activity of 822 ± 13.58 (*P* < 0.05).

### Head Kidney ROS Contents After the Low DO Stress Tolerance Test

Baseline ROS production (OD) for all diet treatments was 0.015 ± 0.001, indicating no significant difference (*P* > 0.05) between ROS production among treatment groups (Figure 6B). Phorbol myristate acetate (PMA) stimulates the head kidney phagocytes of golden pompano, which respond with large reductions in NBT. ROS values in fish fed the D6 diet were significantly lower than that of fish fed the D1 diet (*P* < 0.05). ROS values from phagocytes in fish fed the D6 diet declined sharply compared to the control. However, mean ROS values were not significantly different among D2–D5 diet treatments (*P* > 0.05).

### Fish Eyeball Morphology and Relative Retinal Parameters After the Low DO Stress Tolerance Test

The apparent morphology of a fish eyeball is shown in Figure 6. Fish fed the astaxanthin-supplemented diet had larger black eyeballs than the control fish. Eyes from the fish fed the D1–D5 diets were distinct in anatomical presentation fish fed the D6 diet lacked distinction between the white of the conjunctiva and the sclera from the black eyeball.

**FIGURE 7:**
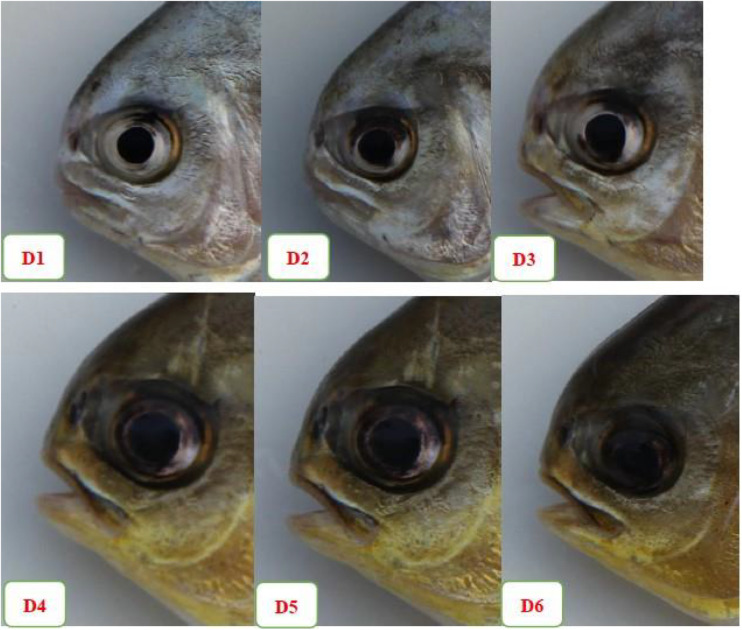
The apparent morphology of fish eyeball among the six experimental diet treatments.

The expression of rhodopsin is shown in Figure 7. Relative rhodopsin mRNA expression levels in the retina of fish treated with the D2–D5 diets were significantly higher than that of fish treated with the D1 diet (*P* < 0.05). However, rhodopsin mRNA expression level in the retina of fish fed the D6 diet decreased to significantly lower level than that found of fish fed the D1 diet (*P* < 0.05).

**FIGURE 8:**
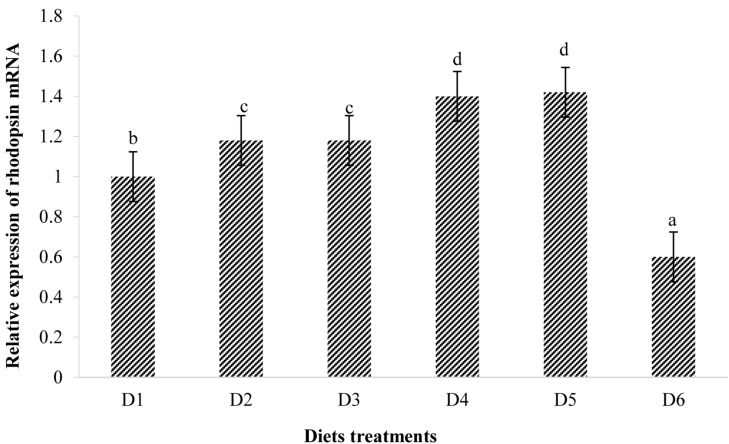
Relative mRNA expressions of rhodopsin in the retina of golden pompano fed the six diets with different astaxanthin levels. Values are means ± SEMs of three replicates, with standard errors represented by vertical bars. ^*a,b,c,d*^ values in the vertical bar with different letters are significantly different (*P* < 0.05). The relative expression of rhodopsin mRNA is as follows: D1, 1.00 ± 0.03; D2, 1.18 ± 0.02; D3, 1.18 ± 0.03; D4, 1.40 ± 0.02; D5, 1.42 ± 0.03; D6, 0.60 ± 0.04.

Retinal expressions of apoptosis-related factors are shown in Figure 8. Relative mRNA expression levels of pBAD, pGSK-3β, and Bcl-xL of fish fed the D2–D5 diets were significantly higher than those of fish fed the D1 diet (*P* < 0.05). However, expression levels of these anti-apoptotic proteins of fish fed the D6 diet were significantly lower than those of fish fed the D1 diet (*P* < 0.05). The expression level of apoptotic protein Bax showed trends opposite to that of anti-apoptotic protein.

**FIGURE 9:**
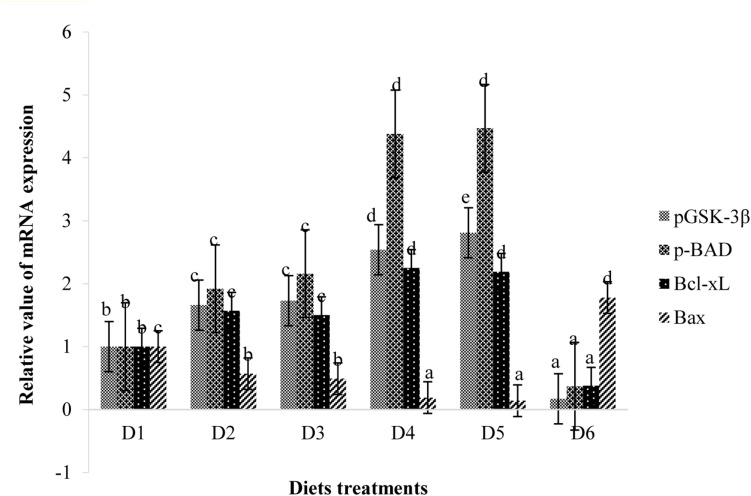
Relative mRNA expressions of pGSK-3β, pBAD, Bcl-xL, and Bax in the retina of golden pompano fed the six diets with different astaxanthin levels. Values are means ± SEMs of three replicates, with standard errors represented by vertical bars. ^*a,b,c,d*^ Values in the vertical bar with different letters are significantly different (*P* < 0.05). The relative expression of pGSK-3β mRNA is as follows: D1, 1.00 ± 0.07; D2, 1.66 ± 0.12; D3, 1.73 ± 0.07; D4, 2.54 ± 0.06; D5, 2.81 ± 0.07; D6, 0.17 ± 0.03. The relative expression of pBAD mRNA is as follows: D1, 1.00 ± 0.05; D2, 1.92 ± 0.13; D3, 2.16 ± 0.09; D4, 4.38 ± 0.09; D5, 4.47 ± 0.07; D6, 0.37 ± 0.02. The relative expression of Bcl-xL mRNA is as follows: D1, 1.00 ± 0.08; D2, 1.57 ± 0.07; D3, 1.50 ± 0.10; D4, 2.25 ± 0.05; D5, 2.19 ± 0.11; D6, 0.38 ± 0.07. The relative expression of Bax mRNA is as follows: D1, 1.00 ± 0.08; D2, 0.57 ± 0.04; D3, 0.49 ± 0.06; D4, 0.19 ± 0.03; D5, 0.14 ± 0.03; D6, 1.78 ± 0.15.

**FIGURE 10:**
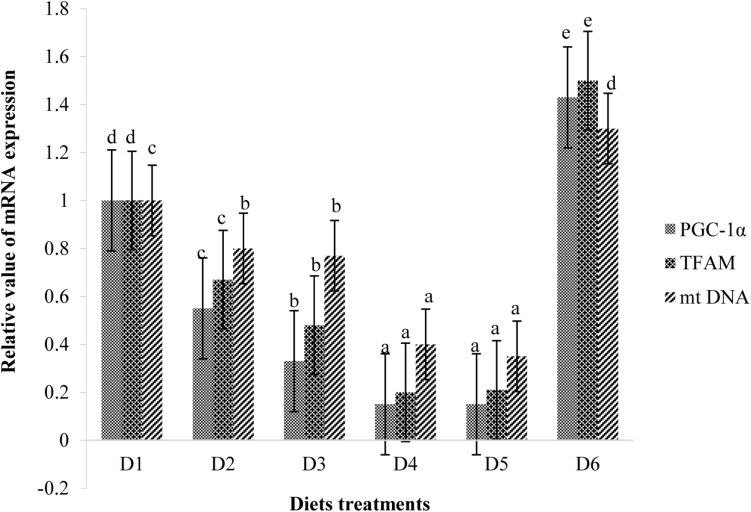
Relative mRNA expressions of PGC-1α, TFAM, and mt DNA in the retina of golden pompano fed the six diets with different astaxanthin levels. Values are means ± SEMs of three replicates, with standard errors represented by vertical bars. ^*a,b,c,d*^ Values in the vertical bar with different letters are significant different (*P* < 0.05). The relative expression of PGC-1α mRNA is as follows: D1, 1.00 ± 0.04; D2, 0.55 ± 0.02; D3, 0.33 ± 0.02; D4, 0.15 ± 0.02; D5, 0.15 ± 0.02; D6, 1.43 ± 0.03. The relative expression of TFAM mRNA is as follows: D1, 1.00 ± 0.06; D2, 0.67 ± 0.04; D3, 0.48 ± 0.03; D4, 0.20 ± 0.07; D5, 0.21 ± 0.02; D6, 1.50 ± 0.08. The relative expression of mtDNA mRNA is as follows: D1, 1.00 ± 0.03; D2, 0.80 ± 0.03; D3, 0.77 ± 0.02; D4, 0.40 ± 0.03; D5, 0.35 ± 0.02; D6, 1.30 ± 0.03.

Retinal mitochondrial function measurements are shown in Figure 9. Relative mRNA expressions of PGC-1α, TFAM, and mtDNA of fish fed D2–D5 diets were significantly lower than those of fish fed the D1 diet (*P* < 0.05). However, expression levels of these transcription factors of fish fed the D6 diet were significantly higher than those of fish fed the D1 diet (*P* < 0.05) (Figure 9). Suitable astaxanthin supplementation (0.005%–0.1%) reduced mRNA overexpression of PGC-1α and TFAM (Figure 10). Moreover, suitable astaxanthin supplementation (0.0005–0.01%) remarkably inhibited up-regulation of retinal mtDNA while excessive astaxanthin supplementation with the D6 diet led to significant increases of retinal mtDNA expression.

## Discussion

### Growth Performance and Immunity

In the present study, no apparent evidence of astaxanthin toxicity to fish was observed. Dietary carotenoid supplementation has been reported to increase the growth performance of fish (Ezhil et al., 2008; Yilmaz and Ergun, 2011; Arulvasu et al., 2013). The point of dietary astaxanthin at 0.049% was estimated to be the levels to provide maximal growth of juvenile *T. ovatus*. The adverse effects of excessive dietary astaxanthin (0.1%) seem to suggest that dosage plays a critical role in growth performance. Moreover, the current immune index may lend insight into the possible effects of feed additives. Blood LZY is a marker that can be used to assess innate immune responses in fish (Saurabh and Sahoo, 2008). LZY hydrolyzes cell walls to kill bacteria, and its activity increases when fish are fed immunostimulants (Burgos-Aceves et al., 2016). Our results from studying suitable astaxanthin level (0.0005–0.01%) administration was consistent with those presented in the aforementioned study. The sharp decline in LZY activity in the highest astaxanthin-supplemented (0.1%) group suggests that excessive astaxanthin destroys the immune system and damages cell membranes of fish, which is consistent with our ROS measurements.

We confirmed that astaxanthin supplementation reduced ROS production, which was consistent with findings presented in *in vitro* studies done with tubular epithelial cells (Kim et al., 2009), murine photoreceptor 661 W cells (Inoue et al., 2017), and Kupffer cells (Li et al., 2017). However, the *in vitro* cell experiments conducted by Kim et al. (2009); Inoue et al. (2017), and Li et al. (2017) could not fully simulate the *in vivo* experiment. Therefore, we speculate from our present results that the distinct actions of ROS under different conditions is due to ROS having dual roles under varying conditions. Our novel data indicate that excessive astaxanthin supplementation under otherwise normal rearing conditions might inhibit ROS production at the physiological level and subsequently lead to mitochondrial dysfunction and oxidative stress. Some physiological levels of ROS may benefit the normal metabolic functions of fish, as described above, although the mechanism remains unknown. This also permits a reference for people taking nutraceuticals involving astaxanthin.

Numerous reports indicate that ROS promote aging processes (Balaban et al., 2005) and are associated with diverse medical disorders, including Alzheimer’s disease, diabetes, cancer, and Parkinson’s disease (Dalle-Donne et al., 2003). To our knowledge, astaxanthin could prevent excessive production of ROS and alleviate oxidative stress under stressful conditions (Niu et al., 2014; Maezawa et al., 2017). Molecular mechanisms of metabolism-induced ROS production, oxidative damage, and metabolic activity related to physiological levels of ROS remain unclear. The study done by Kanazashi et al. (2014) suggests that astaxanthin prevents skeletal muscle capillary regression induced by excessive production of ROS and SOD-1 during hindlimb unloading in rats. Additionally, Jaffer et al. (2015) reported that ROS from mitochondria could promote airway epithelium TGF-β1 expression and activity. Finally, it has been reported that physiological levels of ROS prolong the lifespan of *Helicoverpa armigera* (Zhang et al., 2017). This was also demonstrated from the following morphological changes of fish eyeball and retina.

### Pigmentation, Carotenoid Contents, Carotenoid Retention Efficiency, and Expression Profile of Skin MITFα mRNA and TYR mRNA

Astaxanthin is the most widely used carotenoid pigment additive to aquafeed as it aids in the development of the natural red, orange, and yellow coloration that is highly desired by fish consumers (Storebakken and No, 1992). It has been well documented in previous literature that increasing total dietary carotenoid concentrations leads to incremental increases in average a^∗^ and b^∗^ values with incremental decreases in L^∗^ values of the muscle, skin, and fins of golden pompano (Schubring, 2009; Song et al., 2017). These results also confirmed that the L^∗^ values of filets and skin were negatively correlated to carotenoid contents whereas a^∗^ and b^∗^ values were directly correlated to carotenoid contents (Metusalach, Brown and Shahidi, 1997). Schubring (2009) also found that increased carotenoid concentrations in fish filets and skin led to elevated a^∗^ and b^∗^ values and decreased L^∗^ values. Moreover, our results are consistent with previous findings from studies done on rainbow trout (Rahman et al., 2016). Our results indicated that carotenoid content in the muscle of golden pompano did not further increase when the dietary pigment concentration was above 50 mg/kg (effective content), thereby indicating that 50 mg/kg astaxanthin was sufficient to improve the body color of golden pompano. The lack of response to dietary doses higher than 50 mg/kg has been attributed to reduction in the carotenoid retention efficiency at higher inclusion levels.

Another property related to body pigmentation is relevant gene expression levels. The microphthalmia-associated transcription factor (MITF) gene is expressed in a variety of cell types and has important regulatory roles in neural crest-derived and neuroepithelium-derived pigment cells (Hodgkinson et al., 1993). In amphibians, the MITFα gene, a ventral specific factor, represses differentiation and melanization of melanocytes *in vitro* (Fukuzawa and Bagnara, 1989). The MITFα gene is highly expressed in unmelanized ventral skin and not only suppresses melanization but also appears to promote iridophore localization to this region (Fukuzawa et al., 1995). The TYR gene is a member of the tyrosinase-related protein families. TYR expression and tyrosinase activity have direct effects on eumelanin and pheomelanin expression, thus impacting animal coloration (Kenny et al., 2012). MITFα can regulate the expression of tyrosinase as an important enzyme in melanin synthesis (Lister et al., 2001). MITFα expression is generally higher in dark-colored poultry flesh than in light-colored poultry flesh (Ko et al., 2012). Our current findings related to the MITFα and TYR genes are consistent with the theoretical explanation of Fukuzawa et al. (1995). In this study, MITFα and TYR gene expression levels were closely related to dietary astaxanthin supplementation levels.

### Morphological Changes to the Fish Eyeball

Astaxanthin was found to be highly anti-inflammatory and as effective as prednisolone in reducing *in vitro* production of NO, PGE2, and TNF-α in RAW264.7 cells (Ohgami et al., 2003). Suzuki et al. (2006) reported that astaxanthin may reduce ocular inflammation caused by endotoxin-induced uveitis via down-regulation of pro-inflammatory factors and inhibition of the NF-κB-dependent signaling pathway. Astaxanthin supplementation significantly suppressed production of total hydroperoxides in human aqueous humor (Hashimoto et al., 2013) and inhibited neurotoxicity induced by hydrogen peroxide or serum deprivation in cultured retinal ganglion cells both *in vitro* and *in vivo* (Nakajima et al., 2008). However, we observed that the white part of the eye conjunctiva and the sclera was indistinct from the middle black eyeball in fish from the D6 diet group, which had been administered dietary astaxanthin at a concentration of about 1000 mg/kg. This observation may have been caused by internal bleeding between the conjunctiva and the sclera. Astaxanthin is safe and well tolerated and has no adverse reactions to patients when administered as a component of combined nutraceuticals (Marazzi et al., 2011). No adverse reactions were observed in rats orally administered *H. pluvialis*-produced astaxanthin at concentrations exceeding 2000 mg/kg and 50 mg/kg/day (Takahashi et al., 2005). However, adverse concerns were found in our study of the golden pompano with our use of chemically synthesized astaxanthin, of 10% purity, in dietary concentrations of up to 1%. These effects were not confined only to growth performance and to the eyeball morphology but also to the gut microbial community and gut morphology of the golden pompano. The different effects of astaxanthin may be attributed to differences in astaxanthin sourcing and experimental species studied. The exact reason for the morphological changes of fish eyeball observed in our study is not fully understood and further experiments about the cumulative effects and metabolic mechanism of astaxanthin in the eyes are needed.

### Relative mRNA Expressions of Rhodopsin

The fish retina generates self-sustained oscillations (Falcón et al., 2007). Photoreceptor cells (PCs) contain a biological clock machinery that plays a key role in photoreceptor outer segment disk shedding, visual sensitivity, melatonin synthesis, and expression of genes associated with the opsin family (Falcón et al., 2007; Li et al., 2008). The results of our study suggest that astaxanthin can regulate the abundance of rhodopsin mRNA in the retina of golden pompano. However, the mechanisms involved remain unclear. PC is important in maintaining stability, function, and expression of rhodopsin in the outer segment membrane (Arts and Kohler, 2009), so we explored whether there is a direct relationship between the expression levels of rhodopsin and PC abundance. However, the positive correlation between rhodopsin expression and PC abundance only exists when dietary astaxanthin concentrations were suitable (D1–D5). These results are consistent with the hypothesis that dietary astaxanthin affects opsin-related gene expression in fish and that excessive astaxanthin consumption can adversely affect fish visual function.

### Retinal Expressions of Apoptosis-Related Factors

It is well known that apoptosis is mainly attributed to secondary degeneration of retinal ganglion cells following acute axonal injury (Summers et al., 2014). The PI3K/Akt pathway, as a survival pathway, participates in protection against various stressors. GSK-3β and BAD are downstream substrates of Akt, and their activities are negatively correlated with PI3K/Akt pathway activation (Levkovitch-Verbin et al., 2013). BAD and GSK-3β can activate the mitochondrial apoptotic process, while phosphorylation of BAD and GSK-3β leads to inhibition of caspase-3 activity and apoptosis (Jacobs et al., 2012). In the present study, hypoxic tolerance in fish fed the D1 diet led to apoptosis of retinal neurons and elevated expression of BAD and GSK-3β, thereby activating an endogenous anti-apoptotic response. Appropriate astaxanthin supplementation (D2–D5) inactivated BAD and GSK-3β by promoting phosphorylation, thereby further inhibiting apoptotic processes. Bax is a pro-apoptotic molecule and plays an important role in many apoptotic pathways (Wei et al., 2001). Activated Bax is recruited by mitochondrion to form pores through which pro-apoptotic factors enter the cytosol to induce apoptosis (Desagher and Martinou, 2000). Bcl-xL is an anti-apoptotic molecule located on the outer membrane of mitochondrion and acts to neutralize the action of Bax (Vander Heiden et al., 1997). Bcl-xL can be activated by phosphorylation of BAD and can also be inactivated by dephosphorylation of BAD to form a heterodimer (Yang et al., 1995). The present results showed that suitable astaxanthin diet treatments alleviate cell apoptosis in the retinas under hypoxic conditions, accompanied by up-regulation of retinal anti-apoptotic factors (BAD, GSK-3β, and Bcl-xL) and down-regulation of retinal pro-apoptotic factors (Bax). However, excessive dietary astaxanthin diet supplementation (D6) appears to down-regulate anti-apoptotic factors (BAD, GSK-3β, and Bcl-xL) and up-regulate pro-apoptotic factors (Bax) in the retina. The above results suggest that suitable astaxanthin supplementation may contribute to inhibition of the Bax-mediated mitochondrial apoptotic pathway and maintenance of mitochondrial homeostasis following acute damage.

Mitochondrial dysfunction of retinal cells is found in various types of eye damage (Kong et al., 2009). Changes in ultrastructure and function of mitochondrion were observed in secondary degeneration induced by optic nerve injury (Cummins et al., 2013). To investigate the bidirectional mechanism of astaxanthin in neuroprotection, it is necessary to further evaluate the potential effects of astaxanthin on mitochondrial dysfunction in retinas under hypoxic stress.

PGC-1α and TFAM are nuclear-coded transcription factors that modulate copying of mtDNA (Scarpulla, 2008). We found that suitable dietary astaxanthin treatments suppressed mRNA overexpression of PGC-1α and TFAM in retinas during the low dissolved oxygen trial. PGC-1α is a major upstream transcription factor involved in the expression of many genes required for mitochondrial respiratory function. These transcription factors bind to and transactivate the TFAM promoter region upon transfer to the nucleus (Lin et al., 2002). The main function of TFAM is the up-regulation of mitochondrial genome transcription. Hypoxic stress stimulates mitochondrial biogenesis while also inducing overproduction of ROS, which cause oxidative stress. Therefore, we speculated that suitable dietary astaxanthin could alleviate oxidative stress caused by hypoxia by inhibiting overexpression of PGC-1α and TFAM in the retina. It was also found that suitable dietary astaxanthin treatments (D2–D5) significantly inhibited up-regulation of retinal mtDNA following the low dissolved oxygen trial. However, excessive dietary astaxanthin consumption (D6) during the low dissolved oxygen trial resulted in overexpression of PGC-1α and TFAM, thereby increasing mtDNA transcription in retina. The present results indicate that the neuroprotection of astaxanthin is dose-dependent and that suitable astaxanthin supplementation may be closely related to recovery of mitochondrial biogenesis.

### Gut Microbial Community and Gut Morphology

Lysozyme in fish intestinal mucus contributes to intestinal immunity, health, and microbial community composition (Saurabh and Sahoo, 2008). In this study, 0.0005–0.01% astaxanthin supplementation increased LZY activity, thereby improving fish gut antimicrobial capacity. Moreover, microbial community diversity is critical to well-functioning gastrointestinal systems, thereby influencing fish growth and health. Our results indicate that suitable astaxanthin supplementation (D2–D5 treatment groups) increases the abundance of beneficial bacterial species while reducing the abundance of harmful bacterial species. However, excessive astaxanthin supplementation (D6 treatment group) increases the abundance of harmful bacterial species. Moreover, we found that the ratio of Firmicutes/Bacteroidetes at the phylum level is related to growth. The ratio of Firmicutes/Bacteroidetes is thought to be positively related to mammalian body weight, including that of humans (Thomas and Versalovic, 2010). Increased growth with D2–D5 diet treatment may be attributed to manipulation of intestinal microbial communities by astaxanthin. Nino (2013) reports that dietary immunostimulant supplementation contributes to increases in abundance of *Bacteroides*, *Bifidobacterium*, *Ruminococcus*, *Eubacterium*, and *Lactobacillus*. Immunostimulants can prevent the attachment and colonization of harmful bacteria in the digestive tract (Rodriguez-Estrada et al., 2013), thereby creating opportunities for attachment and colonization of beneficial bacteria, like *Lactobacillus* and *Bifidobacteria*, in the gastrointestinal tract of fish. Our study indirectly correlates suitable astaxanthin supplementation with increased abundance of beneficial intestinal bacteria and excessive astaxanthin supplementation with the growth promotion of harmful intestinal bacteria. We found that the indigenous bacteria Fusobacteria was only present in the D1 diet treatment, demonstrating that astaxanthin supplementation has an ability to manipulate the indigenous bacteria living in the intestine.

Changes in composition of intestinal bacterial communities are also closely related to intestinal morphology. The colonization of harmful bacteria in epithelial tissues is considered essential to infection manifestation in fish (Spring et al., 2000). Inclusion of immunostimulants in feed can effectively reduce colonization of pathogenic bacteria in the intestine and positively contribute to intestinal health (Kim et al., 2011). We found that villus length in D1 diet-treated fish was significantly lower than that from fish fed the D2–D5 diets. The present results indicate that suitable dietary astaxanthin supplementation contributes to the integrity of intestinal morphology and thus improves intestinal immunity. However, the beneficial effects of astaxanthin on intestinal morphology were reduced with excessive supplementation. The precise mechanism for this observation is unknown but is consistent with the observed reduction in pathogenic bacterial colonization to the intestinal epithelium with suitable astaxanthin supplementation while excessive astaxanthin supplementation led to the promotion of pathogenic bacterial colonization to the intestinal epithelium. Additionally, increased length of intestinal villi equates to increased intestinal surface area for nutrient uptake, which facilitates improvement of fish growth. Results of the intestinal morphology and the bacterial community composition studies were consistent with the predicted changes to growth.

## Conclusion

In summary, our research suggests that suitable astaxanthin supplementation influences pigment function by regulating MITFα and TYR gene expression while also exhibiting antioxidant action by reducing ROS levels via improved LZY activity, gut microbial community diversity, and gut morphology. However, high-dose astaxanthin exerted acute injury in neural retina following the low dissolved oxygen trial via down-regulation of anti-apoptotic factors (BAD, GSK-3β, and Bcl-xL) and up-regulation of pro-apoptotic factor (Bax) in retinas. Furthermore, high-dose astaxanthin induced up-regulation of critical mitochondrial component mRNA (PGC-1α and TFAM) and mtDNA copy number. Second-degree polynomial regression of WG indicated that the optimum dietary astaxanthin for juvenile *T. ovatus* is 0.049%.

## Data Availability Statement

The datasets presented in this study can be found in online repositories. The names of the repository/repositories and accession number(s) can be found in the article/Supplementary Material.

## Ethics Statement

The animal study was reviewed and approved by All experimental procedures were conducted in conformity with institutional guidelines for the care and use of laboratory animals in Sun Yat-sen University, Guangzhou, China, and conformed to the National Institutes of Health Guide for Care and Use of Laboratory Animals (Publication No. 85-23, revised 1985).

## Author Contributions

JN designed the study. H-HF and X-SH carried out the rearing work. J-JX and S-YL assisted with sampling. JN, D-QL, WZ, and X-SH tested the samples and analyzed the results. JN wrote the manuscript with contributions from the other authors. All the authors read and approved the final manuscript.

## Conflict of Interest

The authors declare that the research was conducted in the absence of any commercial or financial relationships that could be construed as a potential conflict of interest.

## Footnotes

1 http://qiime.org/

2 http://drive5.com/uparse/

3 http://qiime.org/scripts/alpha_diversity.html

## Supplementary Material

Supplementary Figure 1 Composition and relative abundance of bacterial communities based 16S rDNA sequences. Pattern A, B, C, D, and E indicates the composition and relative abundance of bacterial communities in phylum, class, order, family, and genus level, respectively.

Supplementary Figure 2 **(A)** PCoA, **(B)** multi samples rarefaction curves, **(C)** multi samples Shannon curves and **(D)** relative abundance based on the bacterial community 16S rDNA sequences.
