# Supplementary material for: Dual-Function Analysis of Astaxanthin on Golden Pompano (Trachinotus ovatus) and Its Role in the Regulation of Gastrointestinal Immunity and Retinal Mitochondrial Dysfunction Under Hypoxia Conditions
Source: Front Physiol. 2020 Dec 1;11:568462. doi: 10.3389/fphys.2020.568462 (PMC7736049; doi:10.3389/fphys.2020.568462)
Supplement: Supplementary Figure 1 — Composition and relative abundance of bacterial communities based 16S rDNA sequences. Pattern A, B, C, D, and E indicates the composition and relative abundance of bacterial communities in phylum, class, order, family, and genus level, respectively. [file Table_1.DOCX]

**Table S1** Composition of the basal diet (%)

| Ingredients of basal diet | % |
| --- | --- |
| fish meal ^1^ | 32 |
| soybean meal ^2^ | 30 |
| wheat flour ^2^ | 19 |
| krill meal ^2^ | 3 |
| fish oil ^2^ | 8 |
| Soya lecithin ^2^ | 2 |
| Ca(H_2_PO_4_)_2_ | 2 |
| Premix-vitamine ^3^ | 1 |
| Premix-mineral ^4^ | 1 |
| Choline | 0.5 |
| Vitamin C | 0.5 |
| DL-Methionine ^5^ | 0.4 |
| Lys-HCl (78%) ^6^ | 0.6 |
| Sum | 100 |

^1^ Imported from N.E.L.T.O.Australia Pty Ltd.

^2^Zhuhai Shihai Feed Corporation Ltd, Zhuhai, China

^3^ Vitamin premix (mg or g kg^−1^ diet): thiamin, 25 mg; riboflavin, 45 mg; pyridoxine HCl, 20 mg; vitamin B12, 0.1 mg; vitamin K3, 10 mg; inositol, 800 mg; pantothenic acid, 60 mg; niacin acid, 200 mg; folic acid, 20 mg; biotin, 1.20 mg; retinal acetate, 32 mg; cholecalciferol, 5 mg; α-tocopherol, 120 mg; ascorbic acid, 2000 mg; choline chloride, 2500 mg; ethoxyquin 150 mg; wheat middling, 14.012 g (Niu et al., 2013).

^4^ Mineral premix (mg or g kg^−1^ diet): NaF, 2 mg; KI, 0.8 mg; CoCl2·6H_2_O (1%), 50 mg; CuSO_4_·5H_2_O, 10 mg; FeSO_4_·H_2_O, 80 mg; ZnSO_4_·H_2_O, 50 mg; MnSO_4_·H_2_O, 60 mg; MgSO_4_·7H_2_O, 1200 mg; Ca(H_2_PO_4_)_2_·H_2_O, 3000 mg; NaCl, 100 mg; zoelite, 15.447 g (Niu et al., 2013).

^5^ Imported from Evonik Industries AG

^6^ Imported from NOVUS

**Table S2** Real-time PCR primer sequences.

| Target genes | Primer sequence | Primer length (bp) | Amplicon size  （bp） | References |
| --- | --- | --- | --- | --- |
| β-actin | | | | |
| F | TGCAAAGCCGGATTCGCTGG | 20 | 863 | Xie et al. (2020) |
| R | AGTTGGTGACAATACCGTGC | 20 | 863 |  |
| MITFα | | | | |
| F | CGAAGTACCACATCCAGCAGACCC | 24 | 864 | Liu et al. (2015) |
| R | TTGTCCATGAGCATGTCCTCCAGC | 23 | 863 |  |
| TYR | | | | |
| F | ACTACATGGGGTTCGACTGC | 20 | 865 | Lister et al. (1999) |
| R | GAGCCGTTGTTCATCTGTGA | 20 | 865 |  |
| Rhodopsin | | | | |
| F | CACCTGGGTAATGGCCTGTT | 20 | 863 | Chen et al. (2018) |
| R | CTCCTTGACAGCACAGAGCA | 20 | 864 |  |
| pGSK-3β | | | | |
| F | CCGAAAGGTTTTGCTACCATTCT | 23 | 865 | Zhang et al. (2017) |
| R | AAAATTATTTCCTTTCTGAGCATTCC | 26 | 866 |  |
| BcL-xL | | | | |
| F | CGGGGCACTGTGCGTGGAAA | 20 | 870 | Zhang et al. (2017) |
| R | AGTTGTGGTGGGGGCAGGGT | 20 | 870 |  |
| pBAD | | | | |
| F | TTTATCCATAAGATTAGCGG | 20 | 868 | Zhang et al. (2017) |
| R | AACAGCCAAGCTGGAGACC | 20 | 868 |  |
| Bax | | | | |
| F | ACTTCAACTGGGGCCGCGTG | 20 | 870 | Zhang et al. (2017) |
| R | GAGGCCTTCCCAGCCACCCT | 20 | 870 |  |
| TFAM | | | | |
| F | GAAGCTAAGGGTGATTCACCGC | 22 | 865 | Zhang et al. (2017) |
| R | TGTTGTGGTAGACGCAGAAGG | 21 | 865 |  |
| PGC-1α | | | | |
| F | GTGCAGCCAAGACTCTGTATGG | 22 | 870 | Zhang et al. (2017) |
| R | GTCCAGGTCATTCACATCAAGTTC | 24 | 870 |  |

Xie, J. J., Fang, H. H., He, X. S., Liao, S. Y., Liu, Y. J., Tian, L. X., & Niu, J. (2020) Study on mechanism of synthetic astaxanthin and Haematococcus pluvialis improving the growth performance and antioxidant capacity under acute hypoxia stress of golden pompano (Trachinotus ovatus) and enhancing anti-inflammatory by activating Nrf2-ARE pathway to antagonize the NF-κB pathway. *Aquaculture, 518,* 734657

Liu, H. J., Wen, S., Luo, C., Zhang, Y. Q., Tao, M., Wang, D. W., Deng, S. M., & Xiao, Y. M. (2015) Involvement of the mitfa gene in the development of pigment cell in Japanese ornamental (Koi) carp (Cyprinus carpio L.). *Genetics and Molecular Research, 14 (1),* 2775-2784.

Lister, J. A., Robertson, C. P., Lepage, T., Johnson, S.L., & Ransom, D. W. (1999) nacre encodes a zebrafifish micropthalmia-related protein that regulates neural-crest-derived pigment cell fate. *Development, 126,* 3757-3767.

[Chen](https://pubmed.ncbi.nlm.nih.gov/?term=Chen+JN&cauthor_id=30395593), J. N., [Samadi](https://pubmed.ncbi.nlm.nih.gov/?term=Samadi+S&cauthor_id=30395593), S., & [Chen](https://pubmed.ncbi.nlm.nih.gov/?term=Chen+WJ&cauthor_id=30395593), W. J. (2018) Rhodopsin gene evolution in early teleost fishes. *PLoS One, 13(11),* e0206918.

Wang, R. B., Sun, Q. L., Xia, F. Z., Chen, Z. L., Wu, J. C., Zhang, Y. L., Xu, J. J., & Liu, L. (2017) Methane rescues retinal ganglion cells and limits retinal mitochondrial dysfunction following optic nerve crush. *Experimental Eye Research 159,* 49-57.

**Table S3** Diversity index of gut bacteria of golden pompano fed the experimental diets with different levels of astaxanthin for 8 weeks based on V4 sequences ^1^

| Treatments | D1 | D2 | D3 | D4 | D5 | D6 |
| --- | --- | --- | --- | --- | --- | --- |
| OTUs | 402.3±13.78 | 387.7±12.39 | 391.0±13.05 | 395.0±14.42 | 377.0±5.20 | 375.0±4.58 |
| ACEs | 408.8±15.30 | 397.7±13.44 | 399.4±13.92 | 406.2±13.27 | 386.1±3.91 | 384.5±3.52 |
| Chao 1 | 413.8±18.06 | 401.2±14.50 | 401.6±13.45 | 407.9±13.18 | 389.0±5.32 | 388.3±3.86 |
| Simpson | 0.09±0.01 ^a^ | 0.12±0.01 ^b^ | 0.12±0.01 ^b^ | 0.11±0.01 ^ab^ | 0.11±0.01 ^ab^ | 0.13±0.01 ^b^ |
| Shannon | 3.22±0.04 ^b^ | 2.93±0.01 ^a^ | 2.98±0.02 ^a^ | 3.18±0.05 ^b^ | 2.92±0.06 ^a^ | 2.85±0.05 ^a^ |

^1^ Values are means ± SEMs. *n* = 3. Means in a row without a common superscript letter differ, *P* < 0.05 (one-factor ANOVA, Duncan’s multiple range test). ACE, abundance-based coverage estimator; Chao1, Chao 1 index; OUT, operational taxonomic unit; Shannon, Shannon diversity index; Simpson, Simpson’s diversity index.


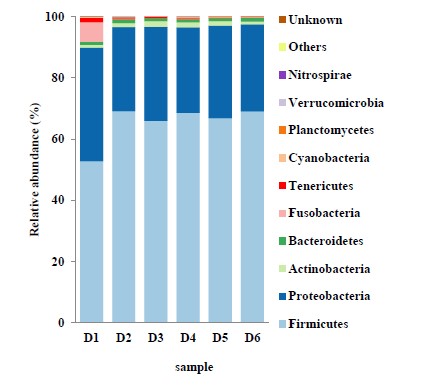
 Fig. S1


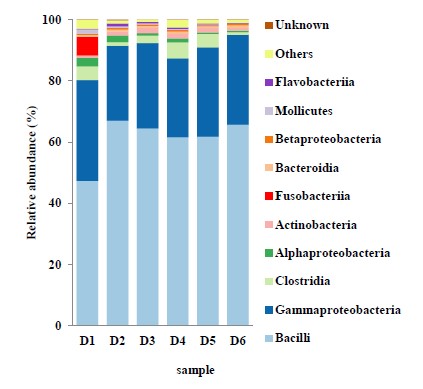


(A) (B)


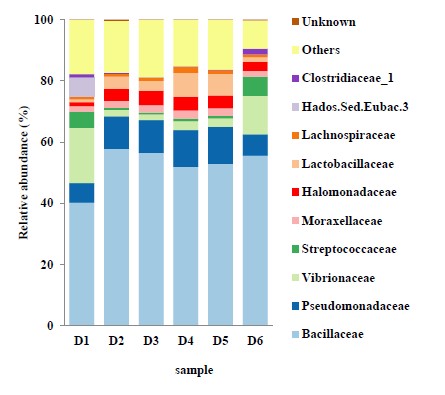

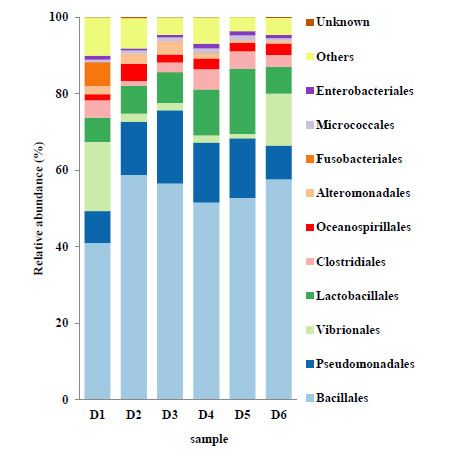


(C) (D)


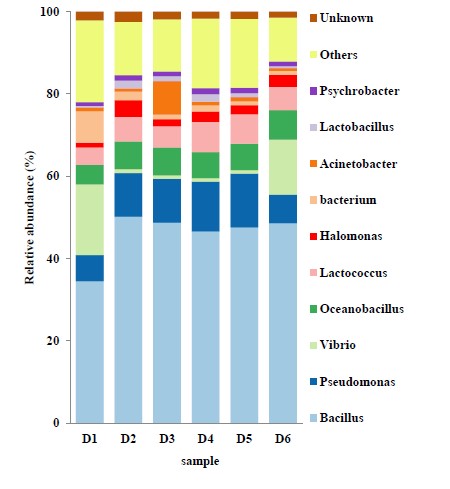


(E)


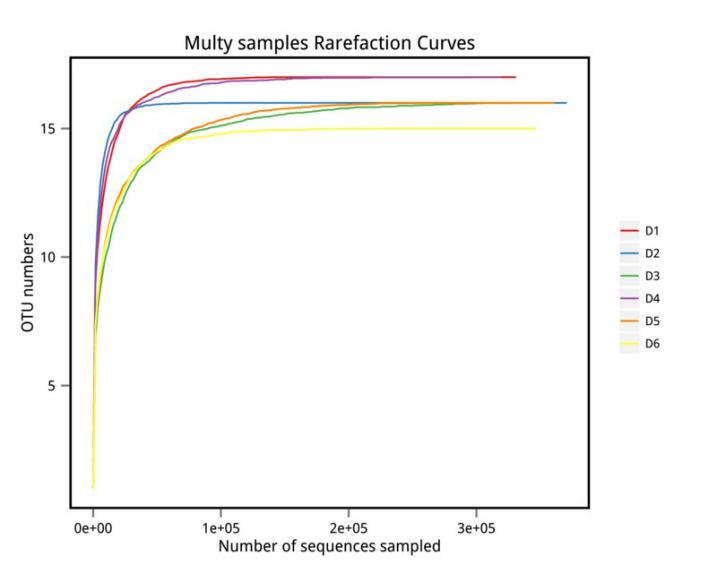

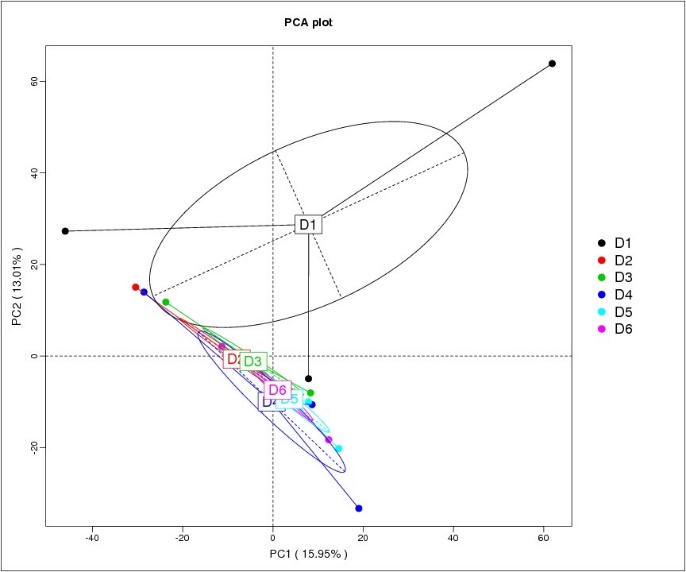
Fig. S2


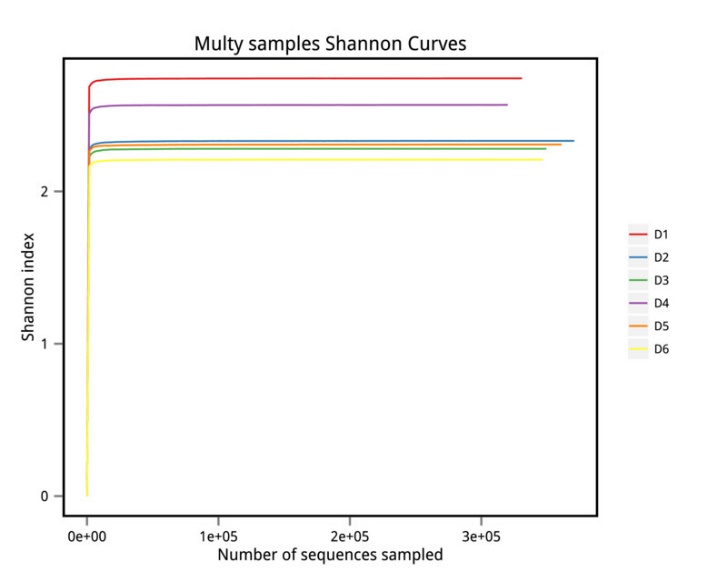

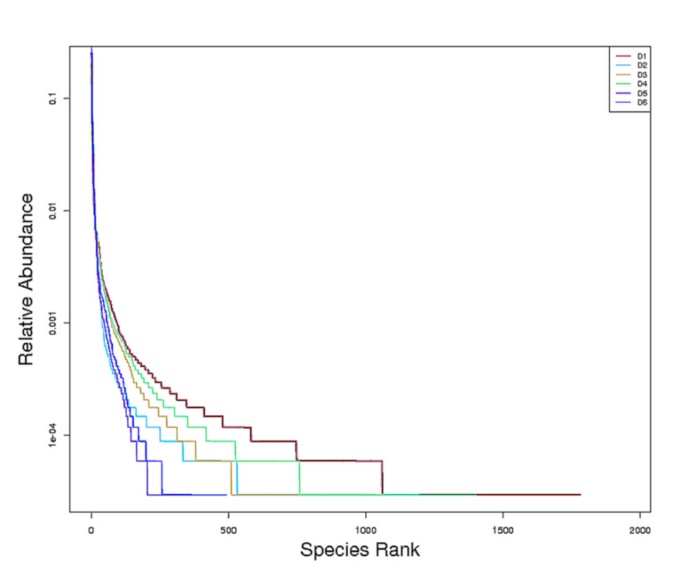
(A) (B)

(C) (D)
